# Supplementary material for: Legionella shows a diverse secondary metabolism dependent on a broad spectrum Sfp-type phosphopantetheinyl transferase
Source: PeerJ. 2016 Nov 24;4:e2720. doi: 10.7717/peerj.2720 (PMC5126622; doi:10.7717/peerj.2720)
Supplement: Supplemental Information 4 — Minimal inhibitory concentration (MIC) [μg/ml] of compounds 4–6 against L. pneumophila, L. longbeachae and L. parisiensis. Mean values of triplicate experiments are shown. [file peerj-04-2720-s004.docx]

**Supplementary Table 4.** Minimal inhibitory concentration (MIC) [µg/ml] of compounds **4**-**6** against *L. pneumophila*, *L. longbeachae* and *L. parisiensis*. Mean values of triplicate experiments are shown.

|  | **4** | **5** | **6** |
| --- | --- | --- | --- |
| *L. pneumophila* | 0.3 | 0.4 | 3.0 |
| *L. longbeachae* | 0.5 | 1.0 | 2.0 |
| *L. parisiensis* | 0.3 | 0.4 | 3.0 |
