# Supplementary material for: Legionella shows a diverse secondary metabolism dependent on a broad spectrum Sfp-type phosphopantetheinyl transferase
Source: PeerJ. 2016 Nov 24;4:e2720. doi: 10.7717/peerj.2720 (PMC5126622; doi:10.7717/peerj.2720)

**Supplementary Figure 1.** Representative examples of clusters O-U from Figure 2. For a full list of clusters, refer to Supplementary Table 3.


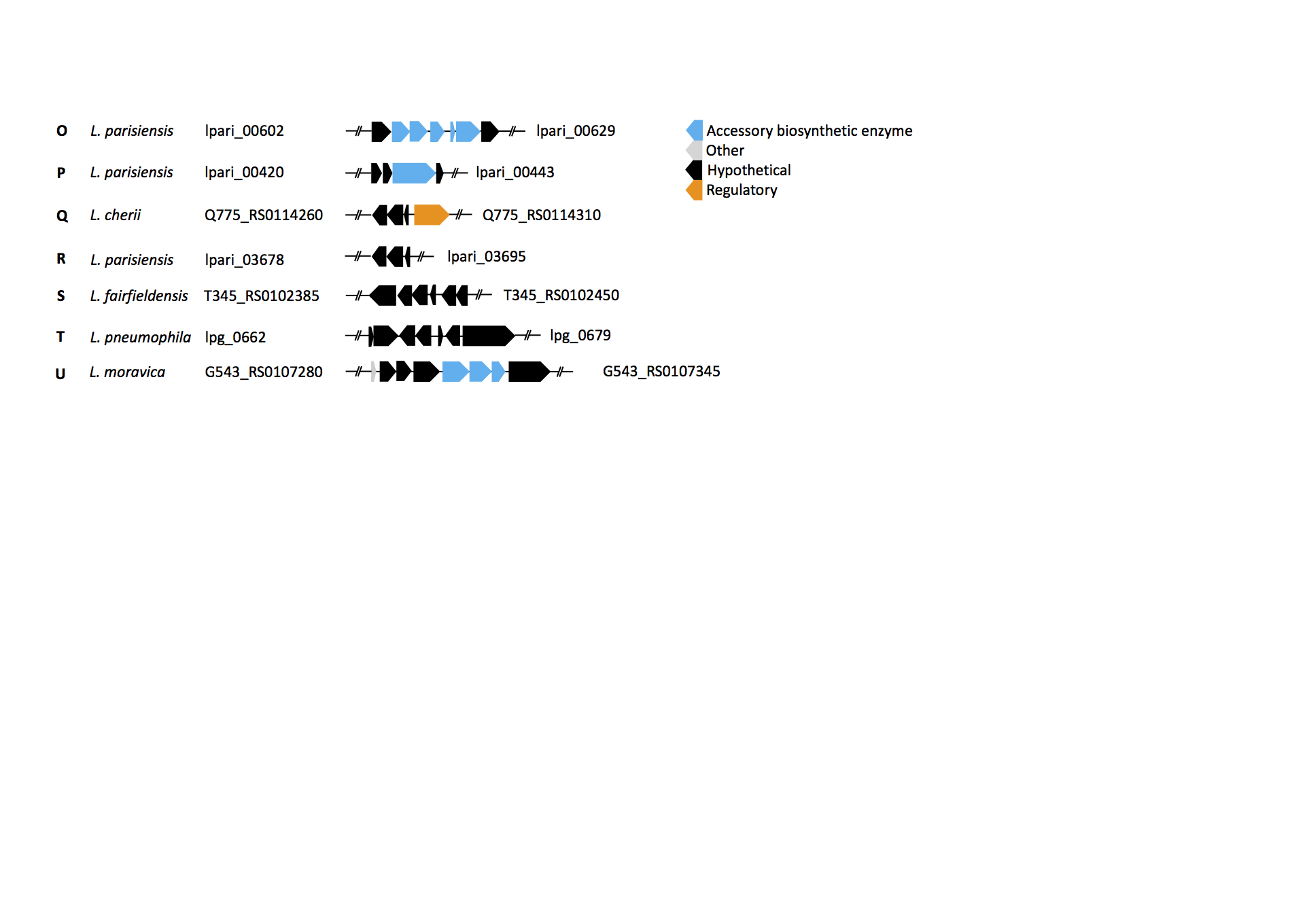

Supplement: Supplemental Information 5 — Representative examples of clusters O-U from Fig. 2. For a full list of clusters, refer to Table S3. [file peerj-04-2720-s005.docx]
