# Supplementary material for: Legionella shows a diverse secondary metabolism dependent on a broad spectrum Sfp-type phosphopantetheinyl transferase
Source: PeerJ. 2016 Nov 24;4:e2720. doi: 10.7717/peerj.2720 (PMC5126622; doi:10.7717/peerj.2720)

**Supplementary Figure 1.** pUC18_indC and pCOLA_LparPPTase were used to transform *E. coli* and subsequently induced with IPTG. Indigoidine production can be seen in (**A**) *E. coli* grown in LB medium and more clearly following (**B**) pelleting of the *E. coli* cultures and resuspension in deionized water.


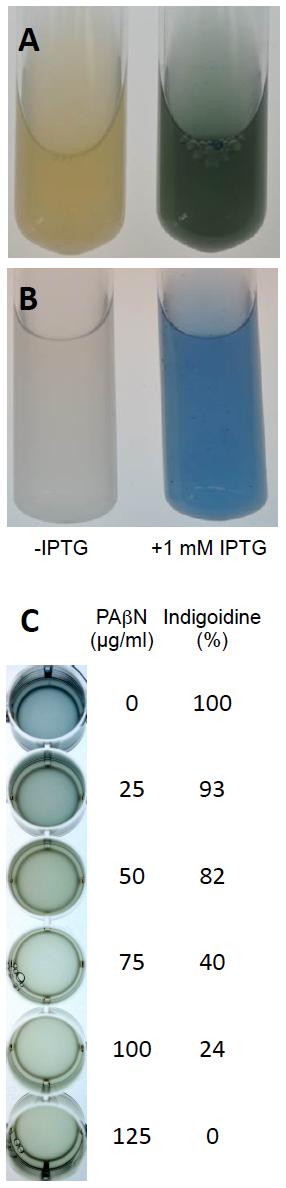

Supplement: Supplemental Information 6 — pUC18_indC and pCOLA_LparPPTase were used to transform E. coli and subsequently induced with IPTG. Indigoidine production can be seen in (A) E. coli grown in LB medium and more clearly following (B) pelleting of the E. coli cultures and resuspension in deionized water. [file peerj-04-2720-s006.docx]
