# Supplementary material for: Legionella shows a diverse secondary metabolism dependent on a broad spectrum Sfp-type phosphopantetheinyl transferase
Source: PeerJ. 2016 Nov 24;4:e2720. doi: 10.7717/peerj.2720 (PMC5126622; doi:10.7717/peerj.2720)

**Supplementary Figure 3**. Selection of unique clusters identified in *Legionella* genomes. The legionellol cluster is shown at the bottom. For a full list of clusters, see Supplementary Table 3. The domain architecture shown (below PKS (red) and NRPS (green) coding sequences) was determined using NCBI’s conserved domain database.


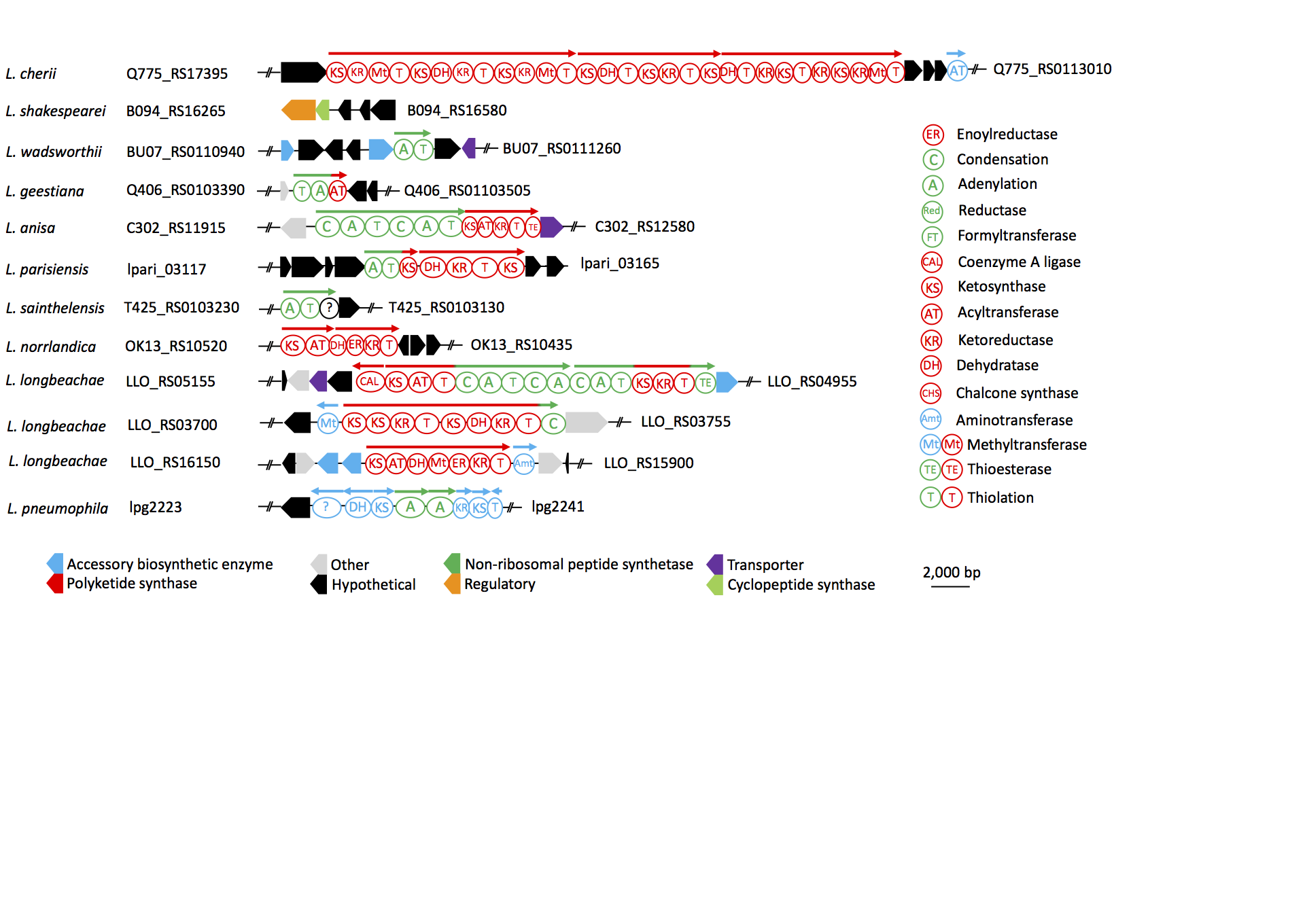

Supplement: Supplemental Information 7 — Selection of unique clusters identified in Legionella genomes. The legionellol cluster is shown at the bottom. For a full list of clusters, see Table S3. The domain architecture shown (below PKS (red) and NRPS (green) coding sequences) was determined using NCBI’s conserved domain database. [file peerj-04-2720-s007.docx]
